# Supplementary material for: A Systematic Pan-Cancer Analysis of MEIS1 in Human Tumors as Prognostic Biomarker and Immunotherapy Target
Source: J Clin Med. 2023 Feb 18;12(4):1646. doi: 10.3390/jcm12041646 (PMC9964192; doi:10.3390/jcm12041646)
Supplement: Supplementary file 1 [file jcm-12-01646-s001.zip › jcm-2203279-supplementary.pdf]

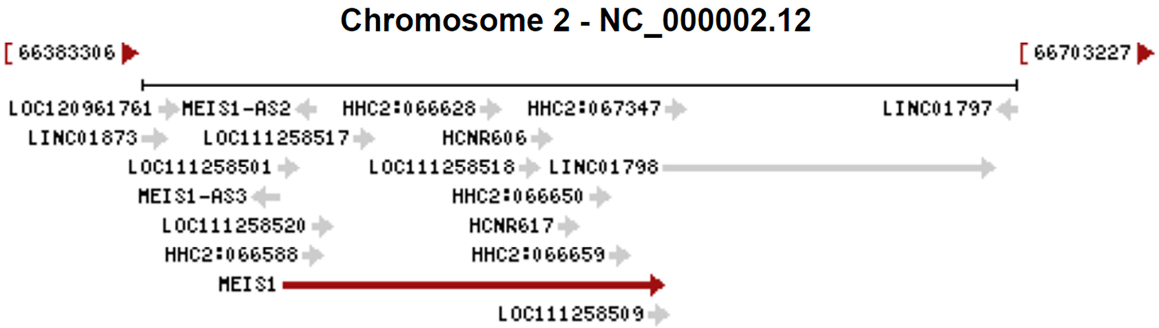

Figure S1. The Genomic context of MEIS1.

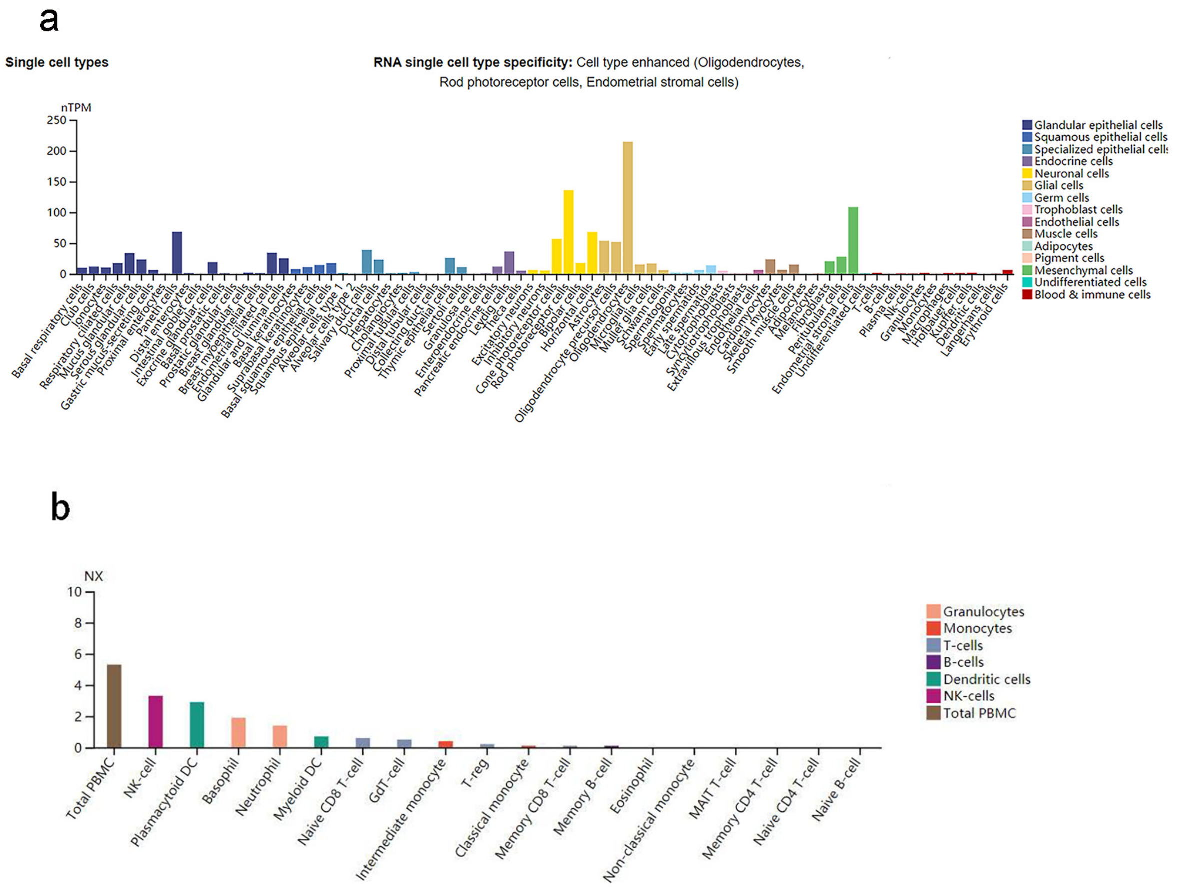

Figure S2. The specificity expression of MEIS1 in single cell. (a) The single cell type spec-ificity expression of MEIS1. (b) The specificity expression of MEIS1 in blood & immune cells.

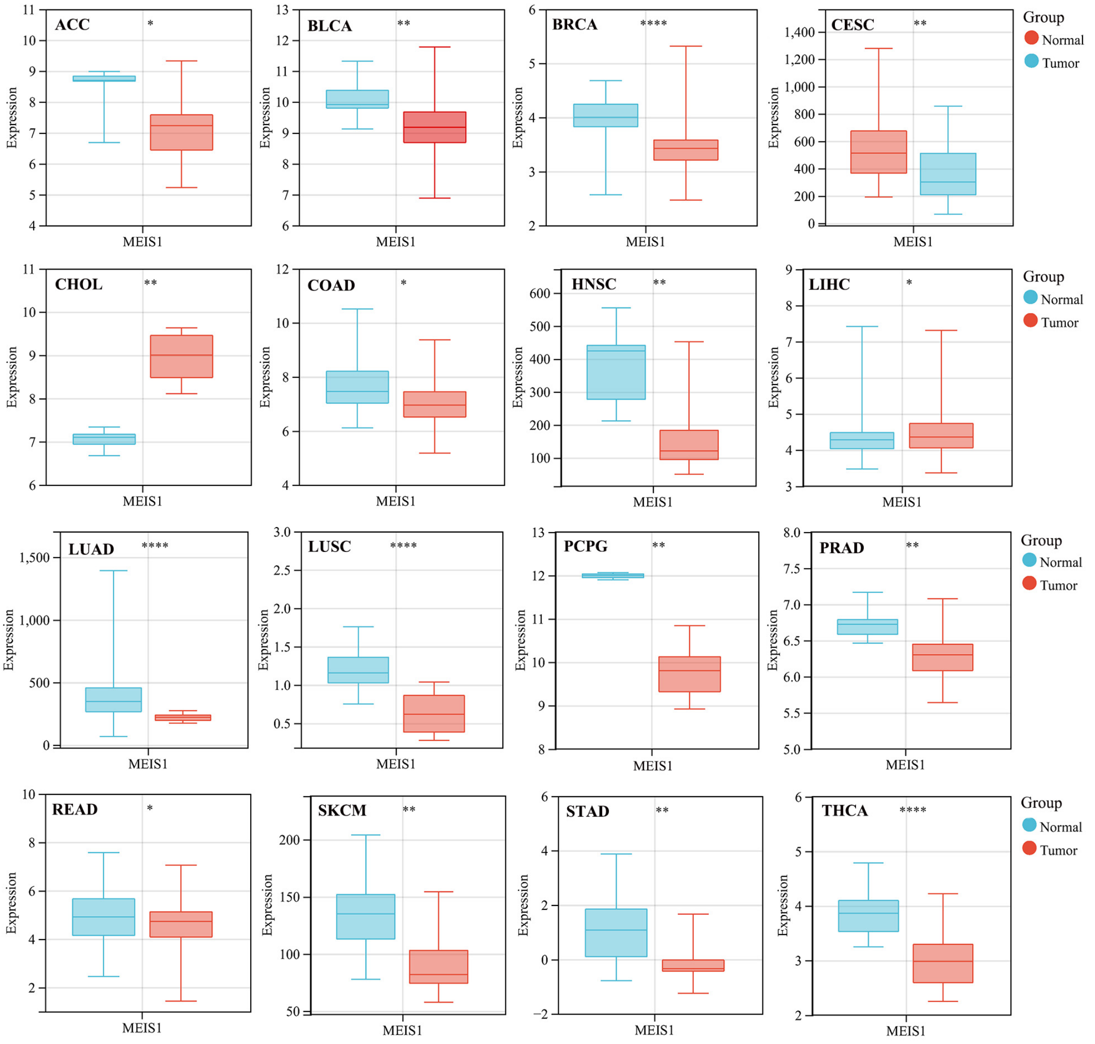

Figure S3. MEIS1 gene expression level in 16 tumor tissues and normal tissues in GEO database. **** P<0.0001; ** P<0.01; * P<0.05.

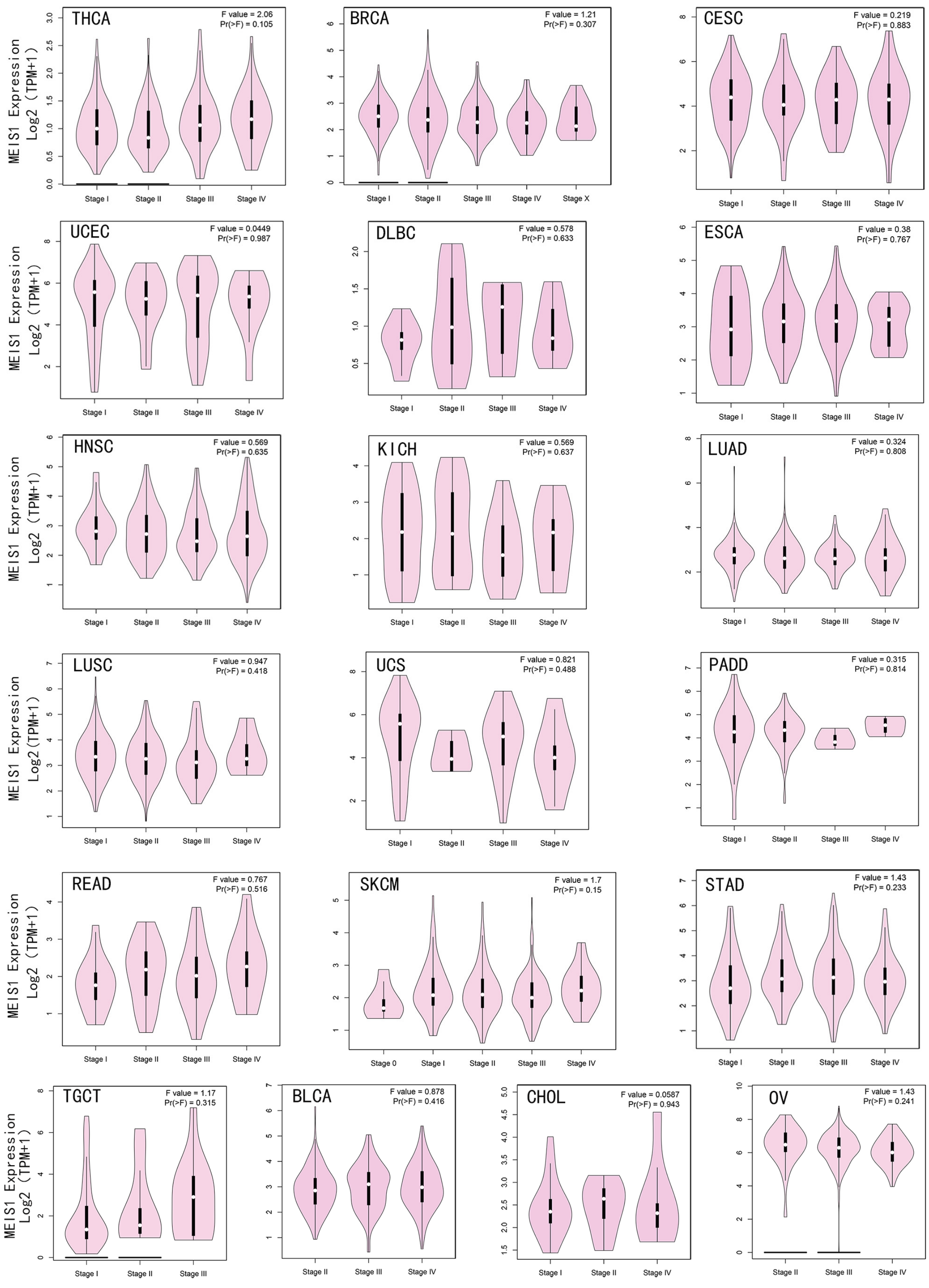

Figure S4. MEIS1 expression level in different pathological stages of THCA, BRCA, CESC, UCEC, DLBC, ESCA, HNSC, Kidney Chromophobe (KICH), LUAD, LUSC, UCS, PAAD, READ, SKCM, STAD, TGCT, BLCA, CHOL, and OV (P>0.05).
